# Disseminated Enteric Salmonella Infection Associated With Empyema and Septic Arthritis in an Immunocompromised Patient

**DOI:** 10.7759/cureus.53392

**Published:** 2024-02-01

**Authors:** Kinnera Urlapu, Vibha Hayagreev, Sujeirys Paulino, Cosmina Zeana, Gilda Diaz-Fuentes, Ravish Singhal

**Affiliations:** 1 Pulmonary Medicine, BronxCare Health System, Bronx, USA; 2 Internal Medicine, BronxCare Health System, Bronx, USA; 3 Pulmonary and Critical Care, BronxCare Health System, Bronx, USA; 4 Pulmonary and Critical Care Medicine, BronxCare Health System, Bronx, USA

**Keywords:** immunocompramized, early interventions, septic complications, thoracic empyema, salmonella infection

## Abstract

Thoracic empyema is a collection of infectious material (pus) in the pleural cavity. Salmonella enterica species rarely cause pleuropulmonary infections. This condition poses a significant challenge in diagnosis and management due to its atypical presentation and potential for severe complications. This is a case of an immunocompromised host with glioblastoma who presented with a large loculated fluid collection in the left pleural space. The patient received broad-spectrum antibiotics and underwent urgent chest tube placement and drainage of pus, which grew Salmonella enterica subspecies enterica. He was also found to be bacteremic with the same organism. Subsequently, he underwent video-assisted thoracoscopic surgery (VATS) with decortication and evacuation of the empyema. Even though the prognosis for empyema is generally unfavorable, with increased morbidity and mortality, due to timely intervention, a successful outcome was achieved in this patient with an atypical presentation of salmonella infection.

## Introduction

Empyema occurs when the pleural space is infected with pus, commonly stemming from bacterial pneumonia. Annually, around one million patients are hospitalized for pneumonia, with 20-40% developing parapneumonic effusion. Of these cases, 5-10% progress to empyema, affecting approximately 32,000 patients annually in the United States, as per the American Association of Thoracic Surgery and American Thoracic Society [1,2]. Empyema bacteriology varies based on community or hospital acquisition. Community-acquired empyema often involves gram-positive bacteria, especially Streptococcus.

On the other hand, hospital-acquired empyema is linked to Staphylococcus aureus, including methicillin-resistant S. aureus (MRSA) and Pseudomonas. Trauma and surgery-related cases commonly involve Staphylococcus aureus. Of note, gram-negative bacteria cause empyema in patients with comorbidities in those with alcohol abuse, gastroesophageal reflux disease (GERD), and diabetes [3]. Pleural empyema due to Salmonella is a rare occurrence; fewer than 40 cases of Salmonella empyema have been reported worldwide [4]. It is most commonly reported in immunocompromised patients or those with underlying diseases such as diabetes mellitus, malignancy, or pulmonary disease [5]. This is a rare case of Salmonella empyema presenting as empyema necessitans in a patient immunocompromised from a coexisting glioblastoma successfully treated with antibiotics, chest tube drainage, and Video-assisted thoracic surgery (VATS).

## Case presentation

A 57-year-old male patient presented to the emergency room with intermittent fevers, dyspnea, and pleuritic chest pain for two days. He lives in the Dominican Republic and traveled to the United States two weeks before the presentation to the emergency room. Initial vital signs were unremarkable except for tachycardia of 102 beats per minute (bpm). On physical examination, he was noted to have decreased breath sounds in the left lung fields. He also had a 5x5 centimeter mass on the left infraclavicular region, which was erythematous and tender to palpation.

**Figure 1:**
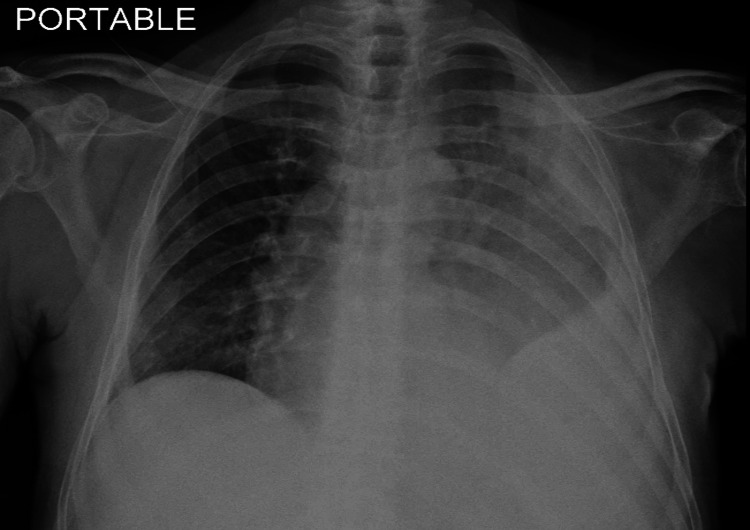
Initial chest-X-ray showing infiltrates in the left lung with moderately large left pleural effusion

**Figure 2:**
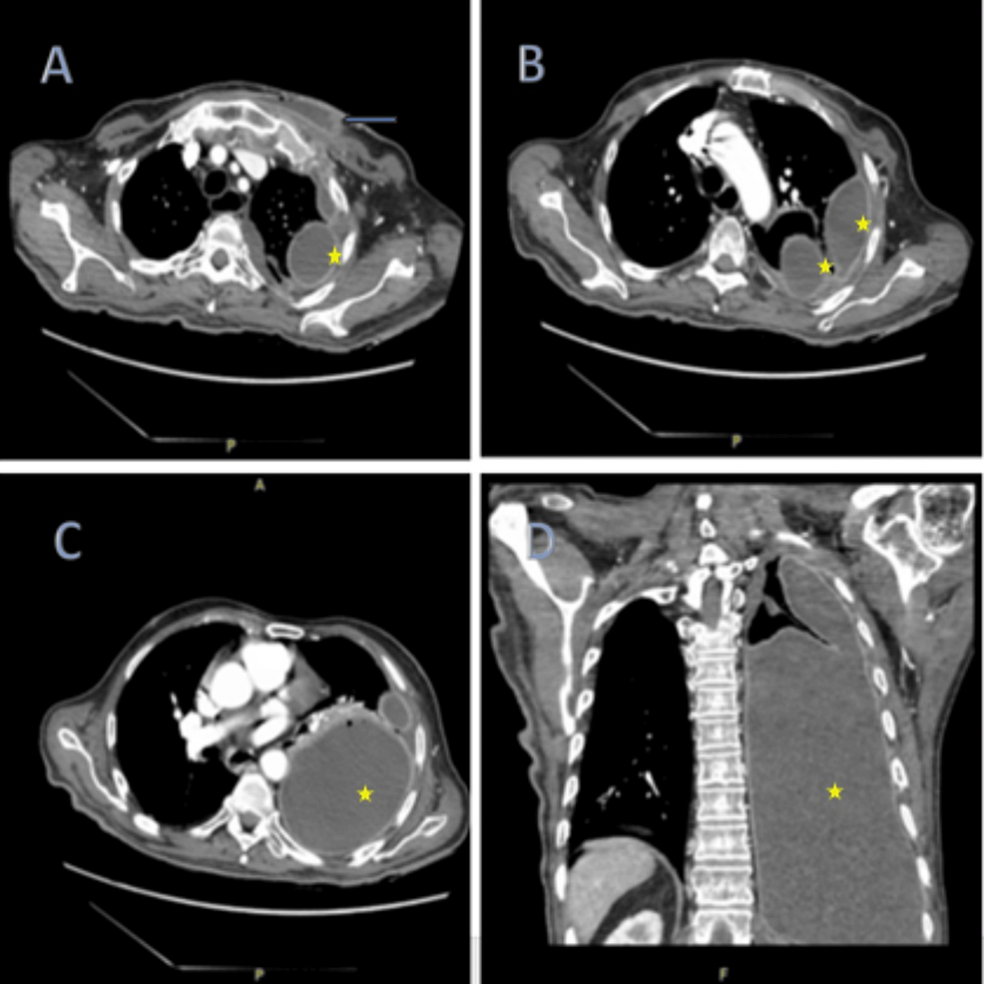
CT chest showing left-sided loculated pleural effusion and soft tissue swelling at the sternoclavicular level. Axial (A, B, C) and coronary (D) planes. Blue arrow: soft tissue swelling on the left chest wall, Yellow stars: loculated pleural effusion

His blood work was significant for mild normocytic, normochromic anemia with hemoglobin (Hgb) of 11.6 g/dl, hyponatremia (130mEq/L), and hypochloremia (94) mEq/L. Lactic acid and inflammatory markers were out of range, as depicted below in Table 1. Chest X-rays and chest CTs showed several abnormalities (Fig 1-2).

**Table 1:** Laboratory parameters at admission

Initial Laboratory Parameters	Results	Reference range
White Blood Cell count	5 k/µL	4.8–10.8 k/µL
Neutrophils	72.7%	40-70%
Erythrocyte sedimentation rate	34 mm/hr	≤30.0 mm/hr
C- Reactive Protein	50.17 mg/L	≤ 5.0 mg/L
Haptoglobin	309 mg/dL	41- 165 mg/dL
Ferritin	1326 ng/ml	12-300 ng/ml
D- Dimer	3128 ng/ml	0-230 ng/ml
Alkaline Phosphatase	139 unit/L	43-160 units/L
Lactate Dehydrogenase	227 unit/L	100-190 units/L
Lactic Acid	3.3 m moles/L	0.5-1.6 m moles/ L

Further history revealed this patient had a significant history of a recent Salmonella liver abscess for which he underwent aspiration and incomplete treatment with antibiotics in the Dominican Republic. The patient also had a history of glioblastoma, which was resected, and he was on radiation therapy. This unusual presentation of chest wall swelling and lower respiratory symptoms raised concern for either a disseminated Salmonella infection with septic arthritis of the left sternoclavicular, left clavicle osteomyelitis leading to thoracic empyema or an empyema necessitans presenting as a chest wall lesion, and clavicle osteomyelitis. 

He was promptly started on broad-spectrum antibiotics, vancomycin, Piperacillin, tazobactam, and Doxycyclin. Thoracic surgery was consulted for chest tube placement to drain sanguinopurulent secretions. The chest wall abscess was also drained simultaneously. Pleural fluid analysis is shown in Table 2.

**Table 2:** Pleural fluid analysis LDH: lactate dehydrogenase

	Pleural Fluid	Reference range
White Blood Cell Count	18 Cells/ mm3	<1000 Cells/ mm3
Segment count	74%	75%
Lymphocytes	21%	23%
RBCs	900 Mil Cells/mm3	<100 Cells/ mm3
LDH	5345 U/dl	<50% of plasma
Protein	3.9 g/dl	1-2 g/dL
Glucose	99 mg/dl	Similar to that of plasma
Adenosine Deaminase	116.1 U/L	<9.2 U/L

Based on the above results, the pleural fluid was exudative in nature according to Light’s criteria. His pleural fluid and blood cultures grew Salmonella enterica species sensitive to ceftriaxone. Other work, including mycobacterial and fungal cultures from the pleural fluid, were negative. Antibiotics were narrowed to ceftriaxone, and metronidazole was added to the regimen. Pleural fluid pathology was negative for malignancy. A repeat CT scan 3 days later showed only slight improvement in the empyema. Due to persistent abscesses collection, the patient underwent VATS with decortication, and an additional 900ml of sanguinopurulent fluid was removed. At the end of the procedure, the lung fully re-expanded, as seen in the post-op X-ray. 

The patient showed clinical improvement, and follow-up blood cultures remained negative; he completed 12 weeks of ceftriaxone at a short-term rehabilitation program with antibiotic infusion services and remained disease-free at a 3-month follow-up. 

## Discussion

Pleural empyema can occur due to complications of bacterial pneumonia, but it can also occur as a complication of thoracic procedures, secondary to chest trauma, esophageal rupture or extension from sources below the diaphragm, and cervical and thoracic spine infections [1,2]. The evolution of empyema is divided into three stages. The first stage, or exudative stage, is represented by acute inflammation with increased capillary permeability leading to neutrophil-rich fluid collection in the pleural space. This stage is followed by a fibrinopurulent stage or stage two, characterized by the bacterial arrival leading to neutrophil accumulation and fibrin deposition. In the second stage, pus is present, and membrane formation occurs, leading to the location or compartmentalization of the fluid. The third and last stage is characterized by organization with the arrival of fibroblasts from the pleural surfaces, forming an inelastic membrane or pleural peel that has the potential to encase the lung and restrict its expansion [2,6].

Patients with empyema can present with cough, dyspnea, fever, and/or chest pain similar to our patient [7]. Rarely, the infection can cause a fistula to the chest wall, named empyema necessitans, as seen in our patient who had a chest wall abscess communicating with the empyema. Imaging studies like X-rays and ultrasound are crucial to diagnosing pleural fluid collection. Contrast-enhanced CT of the chest is a valuable study as it allows for a detailed examination of the lung parenchyma, extension of the pleural fluid, and presence of septations or loculations, helping with treatment decisions [1]. A definite diagnosis is made through pleural fluid analysis with gram stain and culture, which can identify the specific pathogen [1,2,8].

The most commonly identified pathogens are Streptococcus (like Streptococcus pneumoniae and Streptococcus pyogenes), Haemophilus influenzae, Pseudomonas aeruginosa, Mycoplasma, and Staphylococcus aureus [1]. As discussed earlier, the pathogens vary depending on the setting of the infection, such as community-acquired versus nosocomial versus surgery or trauma-related [3]. Salmonella species comprise an extremely rare cause of pulmonary infection and an even rarer cause of empyema, with less than 40 cases reported worldwide to date [4].

Salmonella species was first described by Salmon and Smith in the 1880s [6,9]. It is an intracellular, Gram-negative, non-spore-forming, facultative anaerobic bacilli of the Enterobacteriaceae family. Salmonellae can remain dormant in the reticuloendothelial system and get reactivated via hematogenous spread, and the final clearance of the infection depends on cellular immunity. Salmonella has a tropism for abnormal tissues like malignant tumors, bone infarcts, and aneurysms [9]. Comorbid conditions like acquired immune deficiency syndrome (AIDS), inflammatory bowel disease, malignancy, iron overload, chronic renal insufficiency, diabetes mellitus, alcohol consumption, prolonged corticosteroid therapy, and antineoplastic treatments predispose to extraintestinal Salmonella infection [9]. Our patient with glioblastoma undergoing radiation therapy had positive blood cultures and likely developed pleural empyema due to hematogenous spread to the pleural cavity.

The most common serotypes isolated from Salmonella pulmonary infections are S. enterica serotype Typhimurium and S. enterica serotype Choleraesuis. According to the available literature, S. enterica serotype Enteritidis is much less frequently encountered as a causative agent of respiratory infection than the previous two serotypes. Several crucial factors could precipitate pulmonary infections with S. enterica serotypes; systemic factors such as impaired cell-mediated immunity, impaired B-cell function, prior use of antibiotics, diminished gastric acidity, or low socioeconomic status with poor hygienic conditions; and local factors such as prior lung or pleural disease or abnormalities [10,11]. Other conditions, such as diabetes mellitus, uremia, hypochlorhydria, and gastrectomy, may play a role. However, the real pathophysiological mechanisms remain unclear [10,11].

The management of empyema consists of antibiotic therapy and image-guided pleural drain placement. Other interventions include VATS [2]. Ampicillin, chloramphenicol, and cotrimoxazole were traditionally used to manage non-typhi salmonella infections. However, due to increasing resistance, third-generation cephalosporins are empirically used until further susceptibility to quinolones is available [12]. Pleural empyema or abscess usually requires surgical drainage in addition to antimicrobial therapy [13]. A study reported that intrapleural administration of antibiotics resulted in a sudden increase in the antibacterial activity in the pleural fluid, leading to rapid clinical improvement and eradication of the infection in malignant pleural effusions [14]. Due to the propensity of Salmonella to cause persistent or relapsing infection, a prolonged course of 4 to 6 weeks or longer may be necessary [15]. Due to his immunocompromised state, our patient received a 12-week course of ceftriaxone. The prognosis of empyema remains poor and carries elevated morbidity and mortality [16,17].

## Conclusions

We present a rare case of empyema necessitans due to salmonella enterica. Pleuropulmonary infections due to S. enterica subspecies are very rare. Our case underscores the importance of raising awareness about this unusual presentation of infections with S. enterica subspecies in immunocompromised patients. Successful treatment with antibiotics, drainage, VATS, and aggressive prolonged antibiotic therapy can lead to good outcomes.
